# Distance-based novelty detection model for identifying individuals at risk of developing Alzheimer's disease

**DOI:** 10.3389/fnagi.2024.1285905

**Published:** 2024-04-15

**Authors:** Hongqin Yang, Jiangbing Mao, Qinyong Ye, Magda Bucholc, Shuo Liu, Wenzhao Gao, Jie Pan, Jiawei Xin, Xuemei Ding

**Affiliations:** ^1^Fujian Provincial Key Laboratory for Photonics Technology, Key Laboratory of OptoElectronic Science and Technology for Medicine of Ministry of Education, Fujian Normal University, Fuzhou, China; ^2^Department of Neurology, Fujian Medical University Union Hospital, Fuzhou, China; ^3^School of Computing, Engineering and Intelligent Systems, Ulster University, Derry-Londonderry, Derry, United Kingdom; ^4^Xiamen Jingyi Zhikang Technology Co., Ltd., Xiamen, China; ^5^Fujian Provincial Engineering Research Centre for Public Service Big Data Mining and Application, Fujian Provincial University Engineering Research Centre for Big Data Analysis and Application, Fujian Normal University, Fuzhou, China

**Keywords:** Alzheimer's disease, mild cognitive impairment, novelty detection, decision boundary, decision support system

## Abstract

**Introduction:**

Novelty detection (ND, also known as one-class classification) is a machine learning technique used to identify patterns that are typical of the majority class and can discriminate deviations as novelties. In the context of Alzheimer's disease (AD), ND could be employed to detect abnormal or atypical behavior that may indicate early signs of cognitive decline or the presence of the disease. To date, few research studies have used ND to discriminate the risk of developing AD and mild cognitive impairment (MCI) from healthy controls (HC).

**Methods:**

In this work, two distinct cohorts with highly heterogeneous data, derived from the Australian Imaging Biomarkers and Lifestyle (AIBL) Flagship Study of Ageing project and the Fujian Medical University Union Hospital (FMUUH) China, were employed. An innovative framework with built-in easily interpretable ND models constructed solely on HC data was introduced along with proposing a strategy of distance to boundary (DtB) to detect MCI and AD. Subsequently, a web-based graphical user interface (GUI) that incorporates the proposed framework was developed for non-technical stakeholders.

**Results:**

Our experimental results indicate that the best overall performance of detecting AD individuals in AIBL and FMUUH datasets was obtained by using the Mixture of Gaussian-based ND algorithm applied to single modality, with an AUC of 0.8757 and 0.9443, a sensitivity of 96.79% and 89.09%, and a specificity of 89.63% and 90.92%, respectively.

**Discussion:**

The GUI offers an interactive platform to aid stakeholders in making diagnoses of MCI and AD, enabling streamlined decision-making processes. More importantly, the proposed DtB strategy could visually and quantitatively identify individuals at risk of developing AD.

## 1 Introduction

As the population ages, the impact of neurodegenerative diseases such as Alzheimer's disease (AD), the most common type of dementia (Feigin et al., 2020), is becoming increasingly significant. Neurodegeneration incrementally diminishes the quality of a patient's life and leads to a heavy economic burden in healthcare. In 2022 the Alzheimer's Disease International (ADI) reported that the number of people worldwide suffering from dementia exceeded 50 million, with the total estimated cost of dementia surpassing US$ 1.3 trillion, which is projected to double by 2030 (Gauthier et al., 2022). China, the world's most populous country, accounts for 25% of the world's dementia cases. According to Jia et al. (2020), the total number of people with dementia in China was 14.1 million in 2020, and it is projected to increase to 23.3 million by 2030. The total costs of dementia in China reached US$ 69 billion in 2020 and are estimated to increase to US$ 114.2 billion in 2030 (Ren et al., 2022).

Therefore, there is an urgent need to develop AI-enabled Clinical Decision Support Systems (AI-CDSS) to expedite AD diagnosis and prognosis, thereby enhancing healthcare quality. It has been shown that CDSS can facilitate prompt clinical decision-making processes, minimize medical errors, and lower economic costs. The advancement of AI in healthcare has become a strategic priority in numerous countries, including the US, China, and the UK. For instance, in August 2019, the UK Health Secretary announced a significant investment of £250 million to establish a new national AI lab. This lab aims to tackle major healthcare challenges, including dementia treatments (Hancock, 2019).

As a machine learning technique within the realm of AI, novelty detection (ND) holds the potential for integration into AI-CDSS. ND can detect abnormal behaviors that deviate from typical patterns, making it particularly valuable in safety-critical domains like healthcare. For instance, it has been employed to predict new disease-causing genes (Vasighizaker et al., 2019) and identify anomalous movements in Parkinson's disease patients (Rad et al., 2018). However, there have been limited studies that utilize ND to assess the risk of developing AD. Unlike other machine learning methods, including deep learning applied in the area (Ebrahimighahnavieh et al., 2020; Wang et al., 2020; Liu et al., 2021; Qiao et al., 2022), ND techniques are easily interpretable and applicable even when only one class of data is available (so-called one-class classification), i.e., healthy controls (HC) data in this scenario. A few recent studies of data modeling for mild cognitive impairment (MCI) and AD prediction (Zuo et al., 2021, 2023; Lei et al., 2023) were still built on binary or multiple classes that should be given during the model's training process. They performed binary classification of early MCI (EMCI) vs. HC, late MCI (LMCI) vs. HC, AD vs. HC, EMCI vs. LMCI, and LMCI vs. AD, as well as multiple classifications of LMCI vs. EMCI vs. HC. ND would fully address the issue of training a model based on unbalanced data, which may result in a skewed classification result.

Therefore, this study introduces an innovative approach that holds significant promise for early-stage identification of AD. The main contributions of the work are as follows:

1) Strategic reliance on the power of ND techniques
This novel framework employs a training model that is meticulously constructed solely using the data from HC to uncover anomalous instances, which, in this case, could indicate the presence of early-stage AD. By training the model exclusively on HC data, we taught it to identify deviations from this norm, thus making it exceptionally sensitive to potential signs of cognitive decline even before they manifest clearly.
2) An innovative distance to boundary (DtB) strategy
To enhance the strategic reliance on ND, this study constructed a closed decision boundary tightly surrounding the HC data. Consequently, a distance to the boundary (DtB) strategy is proposed to detect MCI and AD according to the distance of the individual's data point to the decision boundary. Such distance can identify the severity of each individual developing early-stage AD, which in turn can be referred by clinicians for follow-up treatment.
3) Class imbalance alleviation in full
Traditional classification techniques require balanced datasets encompassing both positive and negative classes. However, in the context of AD, we are predominantly concerned with the positive class, i.e., identifying those with the condition. Our framework not only accommodates this skewed focus but thrives in it. By concentrating solely on the HC data for training, we built a model intricately attuned to differentiating healthy patterns from potentially aberrant ones.
4) Cross-regional data employment and an interactive graphical user interface development
We employed two distinct cohorts with highly heterogeneous data from Australia and China, aiming to generalize the proposed DtB strategy. Our findings indicate that a Mixture of Gaussian-based ND method applied to a single modality achieved the highest overall performance in detecting MCI and AD. More importantly, we developed a web-based interactive graphical user interface (GUI) tailored for non-technical stakeholders that incorporates our proposed ND-based framework. This interface introduces a transformative pathway, equipping stakeholders with the means to initiate the evolution of a CDSS that holds the potential to impact AD-related decision-making and intervention strategies.

## 2 Materials and methods

### 2.1 Data extraction

The data used for model development and testing are from the Australian Imaging, Biomarkers and Lifestyle (AIBL) Flagship Study of Aging project (https://www.aibl.csiro.au/) (Ellis et al., 2009) and the Fujian Medical University Union Hospital (FMUUH), China. The usage of both datasets and our submission has been approved by the AIBL Management Committee and the local FMUUH.

#### 2.1.1 The AIBL data

The AIBL data can be categorized into cerebrospinal fluid biomarkers (CSF), cognitive and functional assessments (CFA), magnetic resonance imaging (MRI), positron emission tomography (PET), blood test (BLO), demographic (DEM), and medical history (MH). The CFA involved in our study includes the mini-mental state examination (MMSE), logical memory immediate/delayed recall assessments (LMIR/LMDR), and clinical dementia rating (CDR). The brain imaging data consists of coarse-grained structural MRI and PET with [^11^C]-Pittsburgh compound B (PIB). They are the total volume of gray matter (GM), white matter (WM), cerebrospinal fluid (CSF), and the total number of active pixels (PIB.PET). Table 1 lists the 33 features [4 CFA, 12 BLO, 4 neuroimaging, 2 sociodemographic, 10 medical history (MH), and ApoE genotype features] that were used as potential predictors of cognitive decline associated with AD.

**Table 1:** The AIBL modalities and the corresponding 32 features used in the study.

**Modalities**	**Features**
Cognitive and functional assessments (CFA)	MMSE, LMIR, LMDR, CDR.
Blood analyses (BLO)	Thyroid-stimulating hormone (AXT117), vitamin B12 (BAT126), red blood cell (HMT3), white blood cell (HMT7), platelets (HMT13), hemoglobin (HMT40), mean corpuscular hemoglobin (HMT100), mean corpuscular hemoglobin concentration (HMT102), urea nitrogen (RCT6), serum glucose (RCT11), cholesterol high performance (RCT20), and creatinine rate blanked, (RCT392).
Brain imaging (IMG)	PIB-PET, MRI (GM, WM, CSF).
Demographics (DEM)	Gender, Age.
Medical history (MH)	Psychiatric (MHPSYCH), neurologic other than AD (MH2NEURL), head, eyes, ears, nose, and throat disease (MH4CARD), hepatic (MH6HEPAT), musculoskeletal (MH8MUSCL), endocrine-metabolic (MH9ENDO), gastrointestinal (MH10GAST), renal-genitourinary (MH12RENA), smoking (MH16SMOK), and malignancy (MH17MALI) histories.
ApoE genotype	Two alleles of apolipoprotein genotype (ApoE).

As a gold standard, CDR has been considered a more objective assessment than clinical diagnosis of stage and AD severity. Individuals were categorized into five groups based on the CDR scale levels: the HC (CDR = 0), very mild cognitively impaired (MCI, CDR = 0.5), mild (CDR = 1), moderate (CDR = 2), and severe (CDR = 3) AD patients (Ding et al., 2018). Hence, we used CDR as a target feature due to its strong correlation with clinical diagnostic results (see Supplementary Figure 1). Due to limited data with CDR scores of 2 and 3, we incorporated them and the data with CDR scores of 1 into one category (i.e., AD).

Consequently, we extracted complete non-imaging data from a total of 861 individuals at baseline (BL), where only 262, 222, and 142 individuals had follow-up visits after 18 (M18), 36 (M36), and 54 (M54) months, respectively. Hence, we used 1487 complete non-imaging data, including the CFA, BLO, DEM, MH, and ApoE features, to implement the proposed DtB strategy. However, including MRI and PIB.PET brain imaging data generated only 641 complete data, which were used to construct the ND models and conduct comparative analysis in terms of different modality combinations.

#### 2.1.2 The FMUUH data

To evaluate our proposed strategy on data from different regions, this study also considered 330 local clinical data records (148 HC and 182 AD) obtained from FMUUH China. Six available features containing three cognitive assessments [MMSE, Alzheimer's Disease Cooperative Study - Activities of Daily Living (ADCS-ADL), and Neuropsychiatric Inventory (NPI)] and three demographics (age, education level, and gender) were obtained. The data varied partially from AIBL but were representative since they were provided by our research collaborators and used in the local hospital. Statistical analysis of demographic features in relation to AIBL and FMUUH can be accessed in Supplementary Table 1 and Supplementary Figure 2, respectively.

### 2.2 Feature selection

Min-max normalization was first conducted to assimilate clinical measurements of diverse scales into the range of 0–1. Then, we applied feature selection techniques to identify significant features associated with CDR, which can minimize the computational costs and decrease the analytical complexity. To avoid bias relevant to employing one specific feature selection technique, we adopted in parallel three different filtering approaches based on information gain ratio (IGR) (Karegowda et al., 2010), Pearson's correlation (Grana et al., 2011), and Chi-square (Jin et al., 2006). Afterwards, the Cross-Entropy Monte Carlo rank aggregation algorithm (Pihur et al., 2009) was utilized to aggregate feature ranking results obtained respectively from the above three filters. Finally, the top ten consistently significant features were selected to construct our ND model.

### 2.3 The framework of constructing ND model

**Figure 1:**
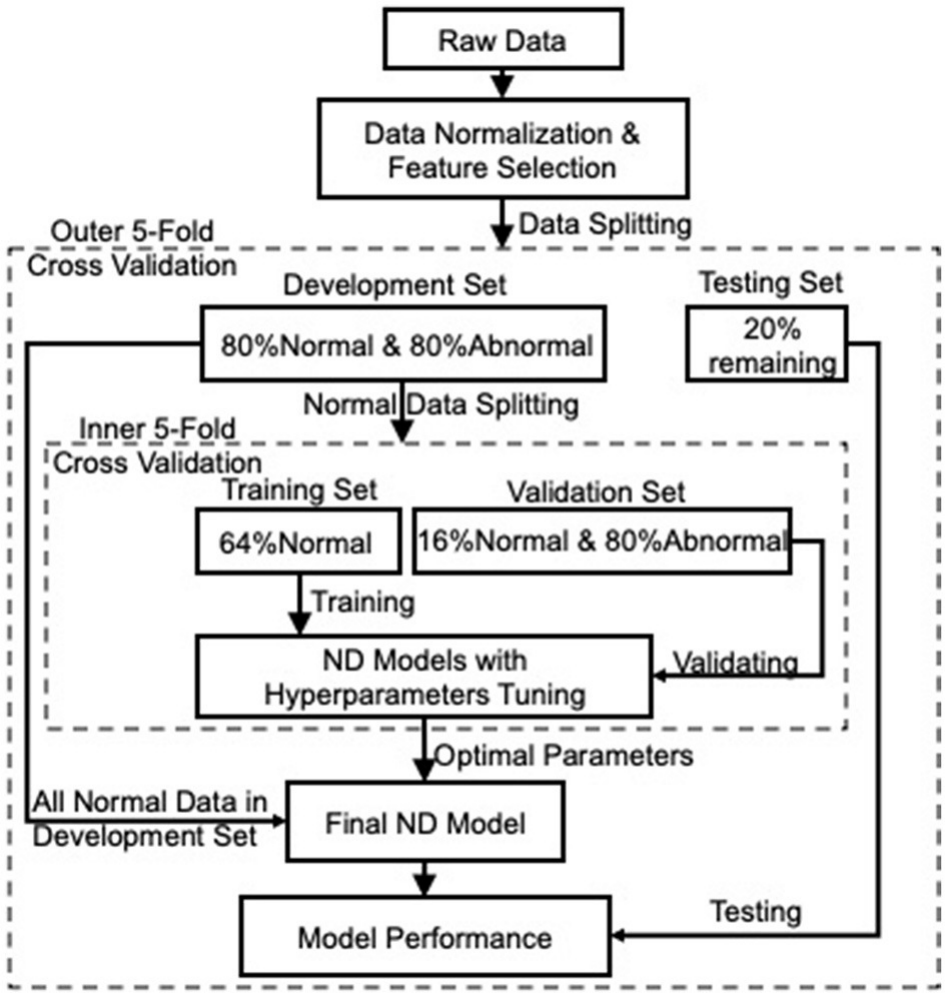
The overall framework of the ND modeling process. Nested cross-validation is employed to optimize models' hyperparameters (inner loop) and select the model (outer loop), which attempts to overcome the problem of overfitting the training set.

The ND model can be constructed with adequate data from the normal class and few from the abnormal class. Consequently, the constructed model has the ability to detect whether unseen data are normal or abnormal according to their fitness to the model. Being different from binary/multi-class classification, where both normal and abnormal data are applied to model training, the ND model is trained solely on normal data, i.e., HC data in this project, as they are much easier to obtain with lower cost than MCI and AD data, hence making our model more robust to unbalanced and unlabeled data. Figure 1 shows the overall framework of constructing the ND model for detecting MCI and AD. A nested cross-validation (NCV) was implemented within the framework, where the inner loop acts for hyperparameters optimization, and the outer loop assesses the performance of the parameters-tuned ND model on the held-out testing set. Such implementation attempts to overcome the problem of overfitting the training set.

The preprocessed data (i.e., data after normalization and feature selection), with respect to each category, were then split into 5-fold: 4-fold for model development (including 80% normal and 80% abnormal data) and 1-fold for the held-out testing (the remaining 20% normal and 20% abnormal). To avoid any bias introduced by random partitioning and to get better repeatability, each one of the 5-fold was selected as the testing set, and the remaining 4-fold were used as the development set (the outer 5-fold CV loop in Figure 1 for model selection). Next, the normal data in each development set were further split into 5-fold for model training and validation. Note that the training set included 4-fold normal data only to construct an ND model, while the remaining 1-fold normal data were combined with the 80% abnormal data, acting as a validation set to validate the trained model. This process was iterated five times (the inner 5-fold CV in Figure 1) to tune the hyperparameters of the applied ND methods. Afterward, the tuned/optimized hyperparameters and the entire normal data in the development set were applied to produce an optimal ND model whose performance would then be assessed on the held-out testing set unseen during the model development process. Finally, the performance of the ND model was averaged over the five outer folds.

### 2.4 Novelty detection methods

Four representative ND methods based on k-nearest neighbor (KNN), Mixture of Gaussian (MoG), KMEANS, and support vector data description (SVDD) were employed in this study. The selection of these methods is due to their comprehensive interpretability, popular applicability in various domains, outstanding historical contributions to ND methods development, and the potential expandability for further research (Ding et al., 2014; Pimental et al., 2014).

#### 2.4.1 K-nearest neighbor

The KNN (Hautamaki et al., 2004) is a representative distance-based ND method assuming that all normal data points are close to each other, and anomalies are far from the normal expectations. The KNN method first calculates the distance between the data point *x* and its *k*-nearest neighbors [denoted as *NN*_*k*_(x)] and then calculates the distance from these nearest neighbors *NN*_*k*_(x) to their *k*-nearest neighbors *NN*_*k*_[*NN*_*k*_(x)]. Finally, it discriminates whether a data point *x* is normal or abnormal by comparing these two distances. The acceptance function, *f*_*KNN*_(*x*), for a test data point *x* can be defined as (Hautamaki et al., 2004):


$$\begin{array}{l} f_{KNN}\left(x\right)=I\left(\frac{\left||x-NN_{k}\left(x\right)|\right|}{\left||NN_{k}\left(x\right)-NN_{k}NN_{k}\left(x\right)|\right|}\leq 1\right) \end{array}$$

where *I*(·) is a logical indicator function. If · is true, then *I*(·) = 1 indicates *x* normal; otherwise *I*(·) = 0 indicates *x* abnormal. ||·|| represents the Euclidean distance. *k* is the parameter to be optimized in KNN. In our experiments, we used the range of integers from 1 to 40 and set step = 1. The max value of the range is usually decided by the size of the data, but the cross-validation results on each fold presented the optimal value, which is always <15.

#### 2.4.2 Mixture of Gaussian

The MoG is a commonly used density-based ND method that calculates a linear combination of multiple components of normal distribution on the given data. The probability density of data *x*, *P*_*MoG*_(*x*), can be estimated with (Bishop, 2006):


$$\begin{array}{l} P_{MoG}\left(x\right)=\frac{1}{N}\sum_{j=1}^{N}\{a_{j}\frac{1}{{\left(2\pi \right)}^{\frac{d}{2}}{\left|\sum j\right|}^{\frac{1}{2}}}\exp\\ \;\left\{-\frac{1}{2}{\left(x-\mu_{j}\right)}^{T}\sum_{j}^{-1}\left(x-\mu_{j}\right)\right\}\} \end{array}$$

where *a*_*j*_ is the mixture coefficients, μ_*j*_ is the mean of the *j*^th^ Gaussian component, is the covariance matrix, and *N* is the number of Gaussian components. Data lying in a high-density area are accepted as normal, while the rest are detected as abnormal. The variable *N* is the parameter to be optimized in MoG. We set *N* in a range of 1–15 with an incremental value of 1.

#### 2.4.3 KMEANS

The KMEANS (Chawla and Gionis, 2013; Gan and Ng, 2017), a representative clustering-based ND method, is one of the most popular techniques due to its simplicity of implementation. This method clusters normal data using a small number (i.e., *k*) of prototypes. The centroids of *k*-clustered prototypes are optimized by the following minimized square error:


$$error_{KMEANS}=\sum_{j}\min_{k}{\left\|x_{i}-\mu_{k}\right\|}^{2}$$

where μ_*k*_ is the centroid associated with the *k*^th^ cluster. Any data excluded by all clusters would be detected as abnormal. *k* is the parameter to be optimized in KMEANS. We set *k* in a range of 1–150 with an incremental value of 1. Again, the cross-validation results on each fold reflected that the optimal value of *k* is always <15.

#### 2.4.4 Support vector data description

The SVDD (Lazzaretti and Tax, 2015) represents an ND method based on a support vector machine. It employs a hypersphere to define a closed decision boundary around normal data. Any data lying outside the boundary is considered abnormal. The general formulation is based on the following relations (Lazzaretti and Tax, 2015):


$$\begin{array}{lll} S & = & \left\{\left(x_{i},x_{j}\right)|x_{i},x_{j}\in same\;class\right\}\\ D & = & \left\{\left(x_{i},x_{j}\right)|x_{i},x_{j}\in different\;classes\right\} \end{array}$$

where *S* is a set of similar examples from the same class, while *D* includes those that are dissimilar from different classes. The learning process involves minimizing the distances between each pair of data points in *S* and maximizing in *D*. The radius of the hypersphere *R* can be calculated by the distance between the center and one of the unbounded support vectors *x*_*S*_:


$$\begin{array}{l} R^{2}=1-2\underset{i}{\sum }a_{i}K\left(x_{i},x_{s}\right)+\underset{i,j}{\sum }a_{i}a_{j}K\left(x_{i},x_{j}\right) \end{array}$$

where *x*_*i*_, *x*_*j*_ are the *i*^*th*^ and *j*^*th*^ data points in the training set. *a* is the Lagrange multiplier with ∑*a* = 1 *and* 0 ≤ *a* ≤ *C*, and *C* is the penalty weight that controls the trade-off between the fraction of rejected normal data and the volume of the hypersphere. In this study, the radial basis kernel *K*, which is the selected kernel, is given by:


$$\begin{array}{l} K\left(x_{i},x_{j}\right)=exp\left(\frac{-||x_{i}-x_{j}|{|}^{2}}{\sigma^{2}}\right) \end{array}$$

where σ represents the kernel parameter (width) to be optimized in SVDD. In our experiments, we set σ in a range of −1.5–1.5 with an incremental value of 0.01 to ensure the model is trained with enough fine granularity.

### 2.5 The algorithm of the proposed DtB strategy

The main idea of the proposed DtB strategy is that the distance of an individual's data point to the ND decision boundary can objectively reflect the individual's severity of developing AD. The theoretical foundation of the strategy is to calculate the distance of each data point to its nearest point on the boundary in order to quantify the severity of cognitive decline. Given the $X=\left\{x_{1},\cdots \;,x_{N}\right\}\;\left(X\in R^{N\times D}\right)$ as the combination of the development dataset *X*^*Dev*^ and testing set *X*^*Test*^, where *N* is the total number of data samples, and *D* represents the dimension of data (i.e., the number of data features), the pseudocode of the proposed DtB algorithm is shown in Algorithm 1.

**Algorithm 1:** The DtB algorithm.

**Input:** Dataset *X*, threshold θ			
**Output:** the decision boundary *B*(·), optimized parameter *K*, and the DtB value			
1:	Randomly split *X* into outer 5-fold, *F*^*Out*^		
2:	**for** each outer fold of *F*^*Out*^ do		
3:		*X*^*Dev*^← 80% HC + 80% non-HC of *X*;	
4:		*X*^*Test*^← the remaining 20% of *X*;	
5:		Randomly split the *X*^*Dev*^ into inner 5-fold, *F*^*In*^	
6:		**for** each inner fold of *F*^*In*^ do	
7:			*X*^*Tr*^← 80% HC in *X*^*Dev*^;
8:			*X*^*Val*^ ← the remaining 20% HC + all non-HC of *X*^*Dev*^;
9:			*B*(·) ← decision boundary constructed on *X*^*Tr*^ by using the four selected ND algorithms;
10:			*K* ← the optimized parameter validated on *B*(·) and *X*^*Val*^;
11:		**end for**	
12:		*FB*(·) ← final boundary constructed on all HC of *X*^*Dev*^, given K;	
13:		$|X^{Test}2FB\left(\cdot \right){|}_{\min}$ = the distance of each $X_{i}^{Test}$ to the closest point *y* on F*B*(·);	
14:		**if** the dot product of the normal vector at *y* and the vector from *y* to the $X_{i}^{Test}$ is less than zero, i.e., *X*^*Test*^ lies inside the boundary	
15:			DtB = ${-|X^{Test}2FB\left(\cdot \right)\;|}_{\min}$; // Indicating the patient is unlikely to develop MCI/AD; the lower the DtB value, the lower the potential of developing MCI/AD;
16:		**else**	
17:			DtB = $|X^{Test}2FB\left(\cdot \right)\;{|}_{\min}$; // The probability and severity of getting the disease will rise with the increase of the DtB value;
18:		**end if**	
19:	**end for**		
20:	Calculate the mean values of sensitivity, specificity, and AUC over 5 folds.		

### 2.6 Evaluation metrics

Three metrics, i.e., sensitivity, specificity, and the area under the receiver operating characteristic (ROC) curve (AUC), were used to evaluate the performance of our ND models. In the context of ND in the medical domain, abnormal and normal data correspond to positive (MCI/AD) and negative (HC) individuals, respectively. The metrics of sensitivity and specificity are defined with:


$$\begin{array}{l} sensitivity=\frac{Number\;of\;true\;positives}{Total\;number\;of\;individuals\;with\;the\;disease} \end{array}$$

$$\begin{array}{l} specificity=\frac{Number\;of\;true\;negatives}{Total\;number\;of\;individuals\;without\;the\;disease} \end{array}$$

Since sensitivity can reliably reflect the correct detection rate of the abnormal data, we considered it as the metric to evaluate the effectiveness of our ND model in correctly detecting all those who have AD. Hence, higher sensitivity is associated with more accurate AD diagnosis. While specificity represents the correct detection rate of the normal data, higher specificity indicates that the novelty detector is less likely to misdiagnose HC. Thus, we chose it as an evaluation metric to correctly identify those who are healthy. Subsequently, AUC is an integrated quantitative presentation of the ROC curve plotted on the sensitivity against the 1-specificity at various thresholds; hence, we adopted it to thoroughly evaluate the overall performance of our ND model.

### 2.7 The interactive GUI development

Using the Shiny package in R (Chang et al., 2022), we developed a user-friendly web-based GUI, an interactive clinical decision support system (CDSS) prototype, based on the proposed DtB strategy. Non-IT users can easily choose different modalities or combinations to build a novelty detector, view the performance of the detector, and further evaluate an individual's severity of developing AD through the visualization of the calculated DtB score and the corresponding CFA scores.

## 3 Results and analysis

### 3.1 Features Importance Ranking Upon the Significance to CDR

To determine if cost-effective and non-invasive AD markers have high discriminative power when they are used for detecting potential AD patients, all features shown in Table 1 were grouped into four modalities: (1) CFA (including LMDR, LMIR, and MMSE); (2) brain imaging features (IMG);(3) medical history and demographics (MH and DEM); and (4) blood tests and ApoE genotype (BLO and ApoE).

**Figure 2:**
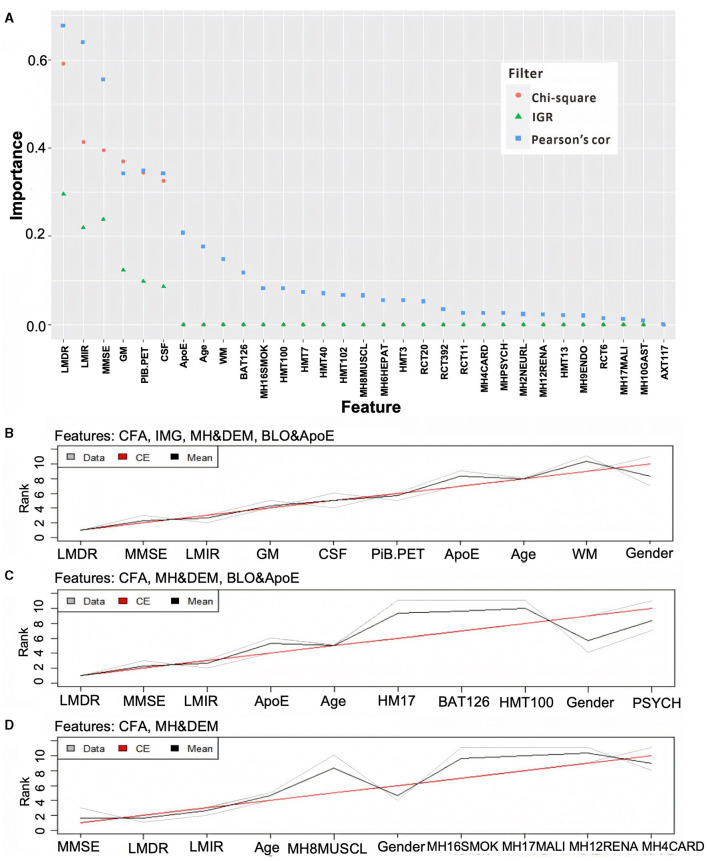
Ranking feature's importance using three different filters **(A)** and aggregating the ranking results associated with different modality combinations **(B–D)**. In **(A)**, the three filters are based on the Chi-square value (orange dot), information gain ratio (IGR, green triangle), and Pearson's correlation (Pearson's cor, blue square). In **(B–D)**, the gray and black lines represent three ranking results and their averages, while the red line reflects the final aggregation result. Note: some Chi-square values are overlapped by IGR as they were all close to zero.

Figure 2A shows that CFA has the strongest correlation with CDR, followed by IMG (excluding WM) and ApoE, then age and WM. In contrast, MH and DEM and BLO modalities are weakly correlated with CDR. The scores of these features' importance are close to zero when applying Chi-square and IGR filters. Different aggregation plans were then carried out to test the ND model performance with/without expensively obtained, invasively tested, and complicated analysis required modalities (e.g., IMG, BLO and ApoE). Figure 2B shows the aggregation results on the feature ranking of all modalities, while Figures 2C, D present those on all modalities, excluding the IMG and BLO and ApoE, respectively. Finally, the top 10 significant features were selected after aggregation. The performance of the ND model trained by all the possible aggregation schemes can be found in Supplementary Table 2.

### 3.2 ND model performance

#### 3.2.1 Model performance on AIBL data

Table 2 compares the AUC, specificity, and sensitivity performance produced by KNN-, MoG-, KMEANS-, and SVDD-based ND algorithms applied to different AIBL modality combinations. It turns out that feature selection significantly improved the performance of MoG, KMEANS, and SVDD. Specifically, the utilization of the CFA modality improved all the models' performance. As a reference, the model performance obtained on all different modality combinations with and without feature selection can be found in Supplementary Tables 2, 3.

**Table 2:** The performance comparison of the ND models constructed by KNN, MoG, KMEANS, and SVDD using different modality combinations of the AIBL data.

**Modality combinations**	**ND algorithms**	**False rejection rate: 0-1**	**False rejection rate** = **0.1**		
		**AUC (95% CI)**	**Specificity**	**Sensitivity**	
			**HC**	**MCI**	**AD**
All modalities without feature selection, i.e., CFA, IMG, MH and DEM, and BLO and ApoE	KNN	0.8479 (0.7897–0.9061)	90.79%	30.25%	38.59%
	MoG	0.7890 (0.6610–0.9169)	82.12%	43.24%	88.47%
	KMEANS	0.7551 (0.6924–0.8178)	67.52%	61.78%	83.57%
	SVDD	0.6856 (0.5604–0.8107)	77.65%	59.26%	73.68%
CFA, IMG, MH and DEM, and BLO and ApoE	KNN	0.8690 (0.8240–0.9140)	89.73%	62.64%	92.82%
	MoG	0.8552 (0.8185–0.8919)	85.89%	64.55%	92.82%
	KMEANS	0.8676 (0.8179–0.9173)	87.43%	58.02%	97.39%
	SVDD	0.8462 (0.7636–0.9288)	88.28%	51.73%	89.54%
CFA, MH and DEM, and BLO and ApoE	KNN	0.8000 (0.7127–0.8873)	89.59%	54.84%	91.89%
	MoG	0.8266 (0.7624–0.8908)	86.04%	59.14%	91.89%
	KMEANS	0.8257 (0.7329–0.9184)	90.59%	52.08%	86.69%
	SVDD	0.8304 (0.6963–0.9644)	86.50%	56.28%	93.80%
CFA and MH and DEM	KNN	0.7858 (0.6710–0.9005)	89.04%	53.68%	86.84%
	MoG	0.8048 (0.6880–0.9214)	89.02%	47.53%	75.43%
	KMEANS	0.7452 (0.6686–0.8217)	84.08%	52.55%	66.72%
	SVDD	0.7783 (0.6646–0.8919)	86.21%	63.93%	83.99%
CFA and IMG	KNN	0.8464 (0.7380–0.9548)	91.40%	68.61%	92.27%
	MoG	0.8558 (0.7717–0.9400)	90.69%	69.86%	95.85%
	KMEANS	0.8564 (0.7492–0.9636)	90.71%	67.60%	96.87%
	SVDD	0.8442 (0.7190–0.9694)	88.99%	68.57%	92.72%
CFA	KNN	0.8521 (0.7250–0.9792)	89.40%	59.87%	96.79%
	**MoG**	**0.8757 (0.7982–0.9532)**	**89.63%**	**67.33%**	**96.79%**
	KMEANS	0.8527 (0.7405–0.9650)	89.11%	56.92%	95.23%
	SVDD	0.8267 (0.7013–0.9521)	84.94%	60.83%	98.43%

False rejection rate (i.e., a tolerant error) identifies the fraction of the HC diagnosed as non-healthy. CI, confidence interval. Bold: The ND algorithm generating the highest AUC score along with the corresponding specificity and sensitivity performance.

Interestingly, models built on CFA only performed better than most of the other modality combinations in the AUC value. Based on the 5-fold CV assessment results, the MoG produced the highest AUC of 0.8757 (95% CI: 0.7982–0.9532) (Table 2, bold), and the KMEANS came next with an AUC of 0.8527 (95% CI: 0.7405–0.9650), followed by KNN and SVDD with AUC of 0.8521 (95% CI: 0.7250–0.9792) and 0.8267 (95% CI: 0.7013–0.9521), respectively. Regarding single-modal features, the MoG model constructed on CFA obviously outperformed those on IMG (AUC of 0.6984, 95% CI of 0.6551–0.7418), MH and DEM (AUC of 0.5938, 95% CI of 0.4076–0.7800), and BLO and ApoE (AUC of 0.5920, 95% CI of 0.5394–0.6446) (see Supplementary Table 3).

Additionally, when using a combination of CFA and IMG modalities, all ND models produced the best detection performance with sensitivity for MCI patients. In particular, MoG presented the highest sensitivity of 69.86%, followed by KNN, SVDD, and KMEANS with a sensitivity of 68.61%, 68.57%, and 67.60%, respectively. Hence, CFA features are the most discriminative, while IMG markers provide supplementary evidence for detecting MCI. Further, adding BLO and ApoE and MH and DEM to the combination of CFA and IMG could not make the model distinguish MCI better and even caused a reduction in the sensitivity of detecting MCI. For example, adding those features made KNN poorer in its sensitivity of MCI, dropping from 68.61% to 62.64%. However, these accessional features could relatively improve the stability of the AUC performance (Table 2). It is worth noting that models built on MH and DEM and BLO and ApoE modalities achieved the worst performance with respect to the AUC, sensitivity, and specificity metrics (Supplementary Table 2).

#### 3.2.2 Model performance on FMUUH data

Consistent with those on AIBL data, experimental results on FMUUH data showed that (Table 3), when applying only CFA for training, MoG again produced the highest average AUC of 0.9443 (95% CI: 0.9037–0.9849) (Table 3, bold), and the KMEANS came next with an AUC of 0.9330 (95% CI: 0.8842–0.9818), followed by KNN and SVDD with AUC of 0.9299 (95% CI: 0.8820–0.9777) and 0.8386 (95% CI: 0.7435–0.9334), respectively.

**Table 3:** The performance comparison of the ND models constructed by KNN, MoG, KMEANS, and SVDD based on the FMUUH data.

**Modality combinations**	**ND algorithms**	**False rejection rate: 0-1**	**False rejection rate** = **0.1**	
		**AUC (95% CI)**	**Specificity**	**Sensitivity**
		**HC**	**MCI**	**AD**
CFA (ADCS-ADL, NPI, MMSE) and DEM (Age, Gender, Education)	KNN	0.9022 (0.8587, 0.9457)	89.13%	88.27%
	MoG	0.8989 (0.8383, 0.9695)	91.01%	89.74%
	KMEANS	0.8869 (0.8332, 0.9406)	88.45%	85.58%
	SVDD	0.7497 (0.7120, 0.7874)	86.89%	79.14%
CFA (ADCS-ADL, NPI, MMSE)	KNN	0.9299 (0.8820, 0.9777)	93.28%	82.15%
	MoG	**0.9443 (0.9037,0.9849)**	**90.92%**	**89.09%**
	KMEANS	0.9330 (0.8842, 0.9818)	92.56%	86.31%
	SVDD	0.8386 (0.7435, 0.9334)	84.75%	82.42%
DEM (age, gender, education)	KNN	0.6921 (0.6654, 0.7188)	66.67%	60.05%
	MoG	0.7223 (0.6335, 0.8111)	72.16%	63.15%
	KMEANS	0.7347 (0.6551, 0.8143)	72.05%	61.58%
	SVDD	0.5128 (0.3778, 0.6478)	71.01%	63.56%

Bold: The ND algorithm generating the highest AUC score along with the corresponding specificity and sensitivity performance.

### 3.3 Decision boundary constructed on HC data only

**Figure 3:**
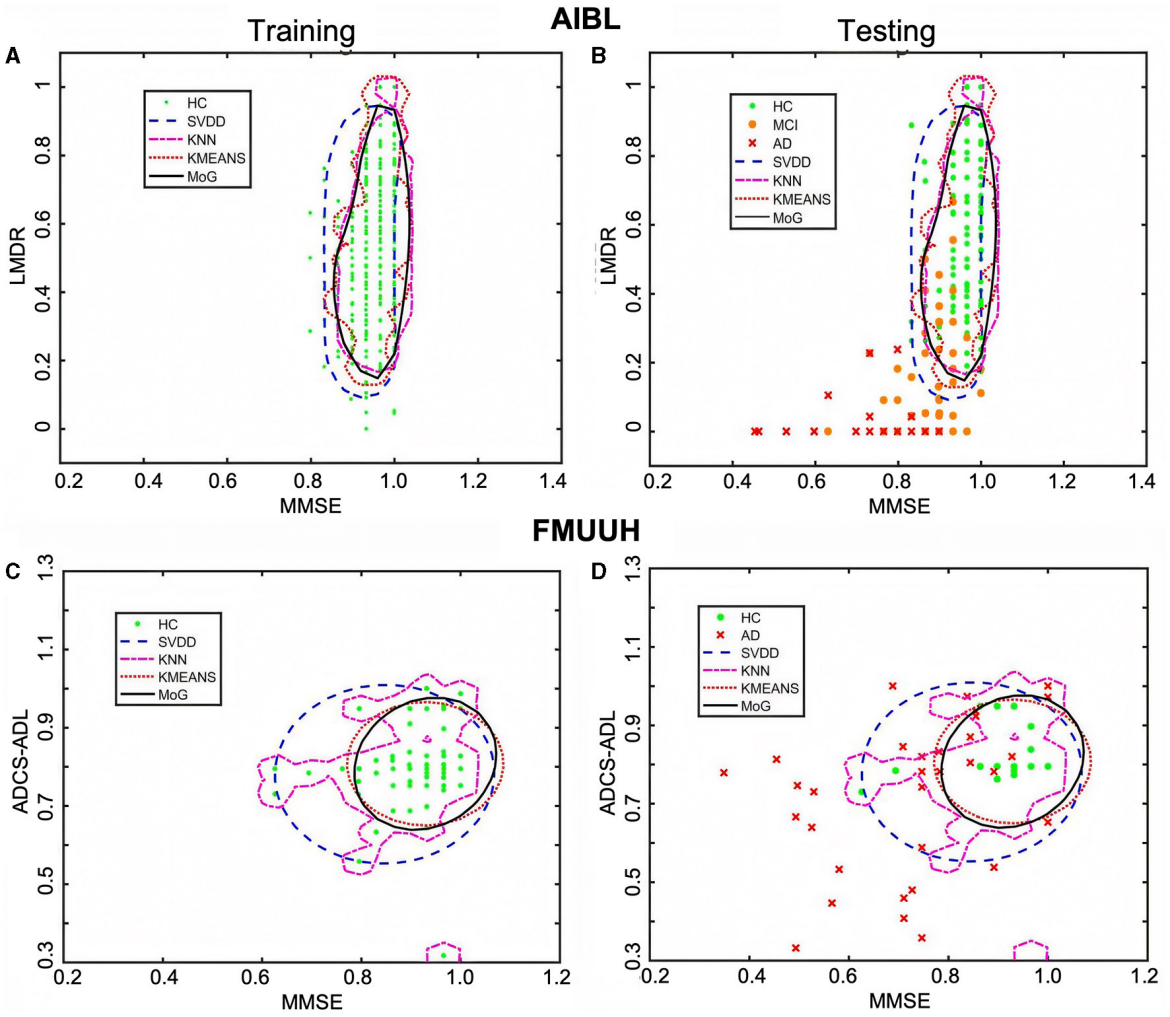
The decision boundaries produced by the four novelty detection methods. **(A, C)** Boundaries trained on HC only in terms of the AIBL and FMUUH data, respectively. **(B, D)** The trained boundaries were used to test the testing data, including both HC and non-HC, with respect to the AIBL and FMUUH data, respectively. The boundaries produced by the SVDD, KNN, KMEANS, and MoG methods are represented by blue dashed, magenta dotted, cyan dotted, and black solid lines, respectively. Green dots, orange dots, and red crosses indicate HC, MCI, and AD. The training threshold (i.e., false rejection rate) is set to 0.1.

Figure 3 illustrates the decision boundary produced by each novelty detector. Randomly, the 1-fold data were selected from both AIBL and FMUUH datasets. The two most important features in the feature selection results (MMSE and ADCS-ADL for FMUUH; MMSE and LMDR for AIBL) were used to visualize the boundary. All selected data were scaled between 0 and 1. To quantify the ND performance on the testing data, Table 4 lists the mean of the 5-fold sensitivity and specificity results for both AIBL and FMUUH, which represent the proportion of non-HC (i.e., MCI and AD) data lying outside the boundary and the proportion of HC data inside the boundary.

**Table 4:** The quantitative evaluation of the visualized decision boundaries constructed by four ND methods (KNN, MoG, KMEANS, and SVDD) using both AIBL and FMUUH data sets.

**Methods**	**AIBL Data**			**FMUUH Data**	
	**Specificity**	**Sensitivity**		**Specificity**	**Sensitivity**
	**HC**	**MCI**	**AD**	**HC**	**AD**
KNN	88.83%	64.88%	98.01%	89.94%	70.59%
MoG	88.72%	67.95%	98.01%	87.99%	76.96%
KMEANS	86.13%	60.32%	99.69%	88.27%	74.93%
SVDD	88.21%	51.96%	97.40%	94.15%	54.41%

Sensitivity for MCI and AD and specificity for HC were calculated based on a 5-fold CV assessment.

#### 3.3.1 Performance of the boundaries produced on AIBL data

All trained boundaries shown in Figure 3A enclosed at least 86% HC data, but the MoG produced a tighter and smoother boundary than others that fit the data distribution best. In terms of the testing results (Table 4), all methods accurately distinguished AD from HC with high sensitivity (higher than 97%); in particular, the MoG boundary rejected 98.01% AD and accepted 88.72% HC. Note that the sensitivity for MCI performed worse than that for AD. Although boundaries generated by the MoG, KNN, and SVDD accepted more than 88% HC, they rejected only 67.95%, 64.88%, and 51.96% MCI, respectively. Hence, the MoG holds a lower misdiagnosis rate for MCI and will be more suitable for early warning and diagnosis of the disease than other methods. Linking to Figure 3B, some MCI data points (orange dots) lie inside the boundaries, indicating some overlap between HC and MCI. This may be because the MCI could not be judged by only two features. Nevertheless, the distance from a data point to the decision boundary can objectively reflect the risk and severity of developing MCI or AD for an individual to a certain extent. For example, most inside MCI points sit close to the decision boundary. From this point of view, the boundary generated by ND methods would be inspiring for solving the problem of clinically unclear diagnostic criteria for MCI. The closer to the boundary that the inside point is located, the more likely the individual presented by the point is developing MCI. On the other hand, the farther to the boundary that the outside point is located, the more likely the individual is getting AD. Therefore, we can benefit from the ND technique and utilize it in early diagnosis and prognosis for MCI and AD.

#### 3.3.2 Performance of the boundaries produced on FMUUH data

On account of the decentralized data distribution, multiple boundaries were generated by the KNN, and one loose boundary was produced by the MoG, KMEANS, and SVDD, respectively (Figure 3C), aiming to include at least 88% HC data. Some overlap between HC and AD (Figure 3D) testing sets reflected high specificity for HC but low sensitivity for AD (Table 4). For example, the MoG obtained the lowest specificity of 87.99% for HC but the highest sensitivity of 76.96% for AD. Therefore, we proposed a Distance to Boundary (DtB) strategy to address this inevitable low-sensitivity issue caused by the overlap between HC and non-HC (MCI/AD) and detect potential MCI/AD further.

### 3.4 The DtB strategy

**Figure 4:**
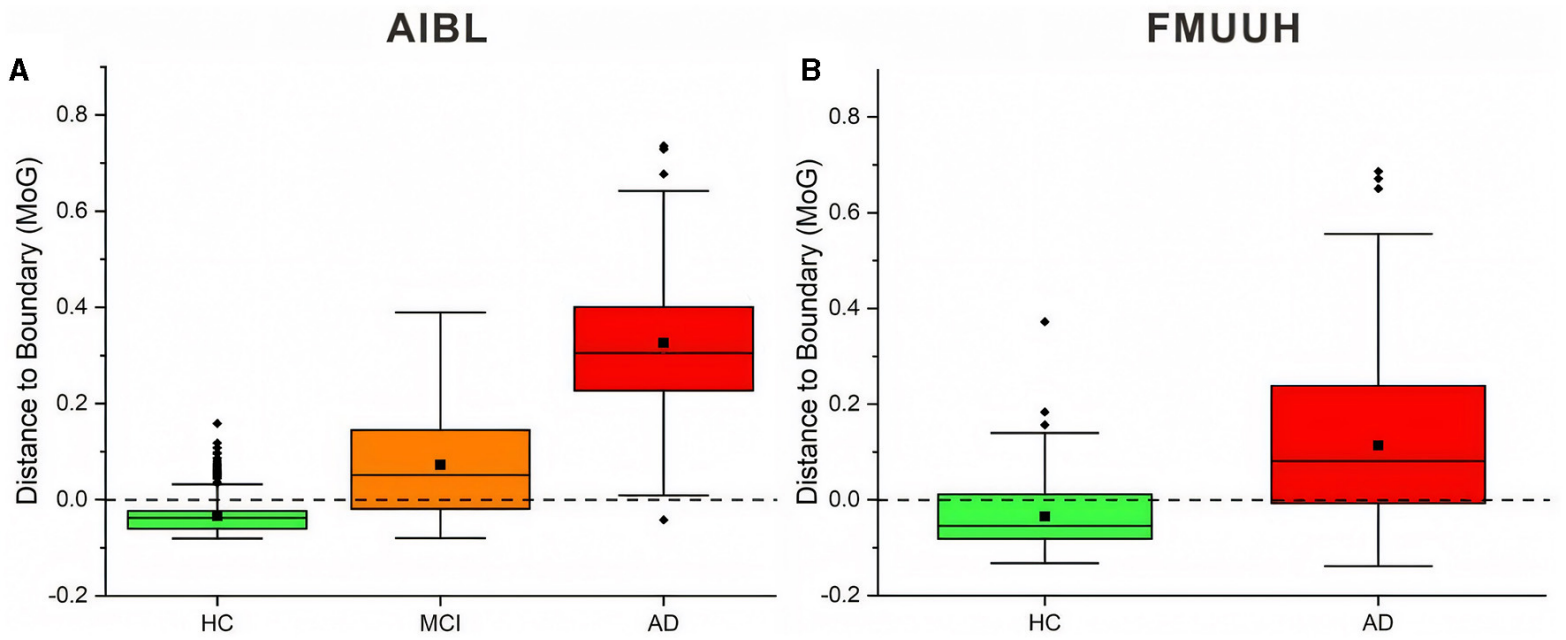
Boxplots of the distance between AIBL and FMUUH data points and ND boundary generated by MoG. Points beyond 1.5 times IQR (interquartile range) are considered outliers of the boxplot, represented by a solid diamond. The solid square is the mean value, and the dotted line (i.e., the DtB value of 0) is the ND boundary location. **(A)** for the AIBL data and **(B)** for the FMUUH data.

To better describe the strategy, we chose the MoG algorithm, which generated stable closed boundary precisely surrounding the HC data and the best overall performance, to calculate the DtB values on testing data. The DtB calculation was carried out on the 5-fold CV assessment results. Figure 4 depicts the boxplots for the distance of each categorical (i.e., HC, MCI, and AD) data points to the decision boundary constructed by MoG (Figure 4A for AIBL and Figure 4B for FMUUH). We define the sign for the distance of inner data to the boundary as negative, while that for outer data is positive. Table 5 lists the descriptive statistics of the boxplots shown in Figure 4.

**Table 5:** Descriptive statistics of DtB scores.

**Data**	**Classes**	**Mean**	**Mode**	**Minimum**	**Median**	**Maximum**
AIBL	HC	−0.0342	−0.0665	−0.0806	−0.0377	0.1587
	MCI	0.0725	0.2587	−0.0800	0.0511	0.3890
	AD	0.3258	0.3052	−0.0420	0.3045	0.7356
FMUUH	HC	−0.0348	−0.0813	−0.1324	−0.0545	0.3722
	AD	0.1136	0.0813	−0.1384	0.0813	0.6860

Figure 4A reveals that the first and the third quartiles, median and mean, and maximum DtB values of AD are higher than those of MCI, which in turn are higher than those of HC. Additionally, the boxes of MCI and AD are more than twice as long as the HC box. The overall spreads are quite different, and some overlaps occur among the three categories. Since there are more outliers, which would affect calculations of skewness, the boxplot for HC shows some slight bottom-skew compared with the main bodies of data for MCI and AD, which have symmetric appearances. Consequently, the potential outliers of HC probably indicate the risk of getting MCI, while the outliers of MCI may present the possibility of developing AD, and the outliers of AD are associated with more severe disease development. Overall, the HC, MCI, and AD categories do vary with the DtB values. Figure 4A could interestingly visualize our proposed DtB strategy. For example, data points lying outside the boundary might represent patients who are at greater risk of developing AD, as the distance from the boundary increases. On the other hand, the inner data points that are nearer to the boundary might indicate a higher risk of cognitive decline. The mean locations of HC, MCI, and AD data against the boundary can be found in Supplementary Figure 3.

Basically, the DtB boxplot of FMUUH data shows a similar trend to that of AIBL data. The AD box is longer than the HC's, which presents higher first and third quartiles, median, mean, and maximum DtB values than those of HC (Figure 4B). The difference is that the AD box of FMUUH data is slightly across the ND boundary (the horizontal dotted line), which is similar to the MCI box of AIBL data. Although an AD data point may be misdiagnosed as HC due to its lying inside the boundary, we can still detect its risk of developing AD according to its DtB value. Note that, however, different features used in Figures 3A–D, as well as different population cohorts from Australia and China, may result in the difference between Figures 4A, B. Figure 4B reveals that some MCI patients might be included in the AD category, which also reflects an urgent need to integrate various data resources acquired from different departments of the local hospital. This concern has been communicated and confirmed with our research collaborators in the hospital.

Table 5 reflects that the minimum DtB values of HC and MCI in AIBL are very close (i.e., −0.0806 and −0.0800). The reason could be a certain degree of overlap between HC and MCI, as well as the lower boundary dimension and the ambiguity of cutoff scores for determining MCI (Pandya et al., 2016). In a higher-dimensional feature space, the DtB strategy can integrate more different assessment indicators and criteria to describe the severity of MCI patients more precisely. Similarly, the closed minimum DtB values of HC and AD in FMUUH data (i.e., −0.1324 and −0.1384) also reflect the aforementioned hint.

### 3.5 The user-friendly interactive GUI for CDSS

**Figure 5:**
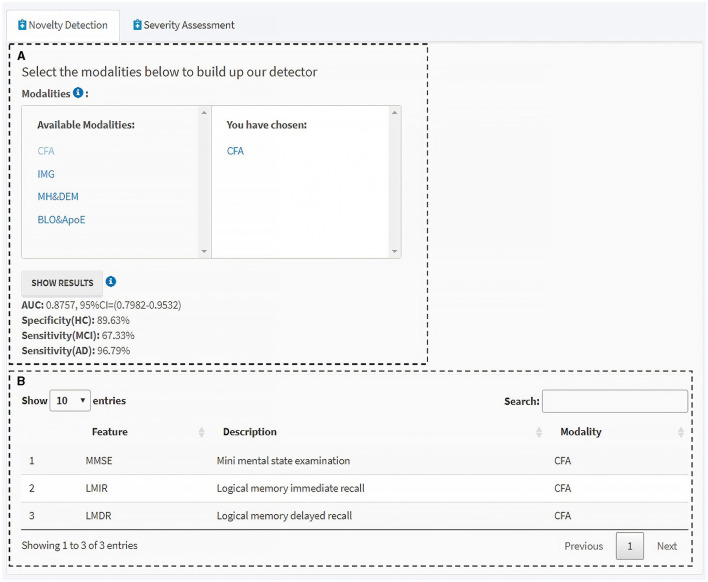
Novelty detection module of the interactive GUI for CDSS. **(A)** Available modalities are provided in the left panel, and those chosen by users in the right. Clicking the “SHOW RESULTS” button lists the performance of the novelty detector constructed using the MoG-based ND algorithm on the selected modality/modalities. **(B)** Information table of the features and the corresponding descriptions within the selected modality/modalities. Additional information can be found through the info icons.

To employ and translate our proposed DtB strategy for non-IT users, especially for clinicians, we extended our work by developing a user-friendly web-based interactive GUI, which is made available at: https://ad-cdss.shinyapps.io/cdss/. By clicking the web link, users are first brought into the Novelty Detection module (Figure 5), where a multiple-choice option of available modalities is provided in the top-left panel, and the chosen modality/modalities is then shown in the top-right panel (Figure 5A). Subsequently, the performance of the novelty detector constructed on the chosen modality (or a combination of chosen modalities) will be presented in terms of AUC, specificity for HC, sensitivity for MCI, and AD metrics once users click the SHOW RESULTS button (Figure 5A bottom panel). For computational efficiency, the GUI APP uses the MoG-based ND algorithm, which has been demonstrated to have the best AUC performance (Tables 2, 3). For users' information, data features and the corresponding descriptions in relation to the selected modality/modalities are listed at the bottom panel (Figure 5B). Additional information can be found in the info icons (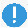).

**Figure 6:**
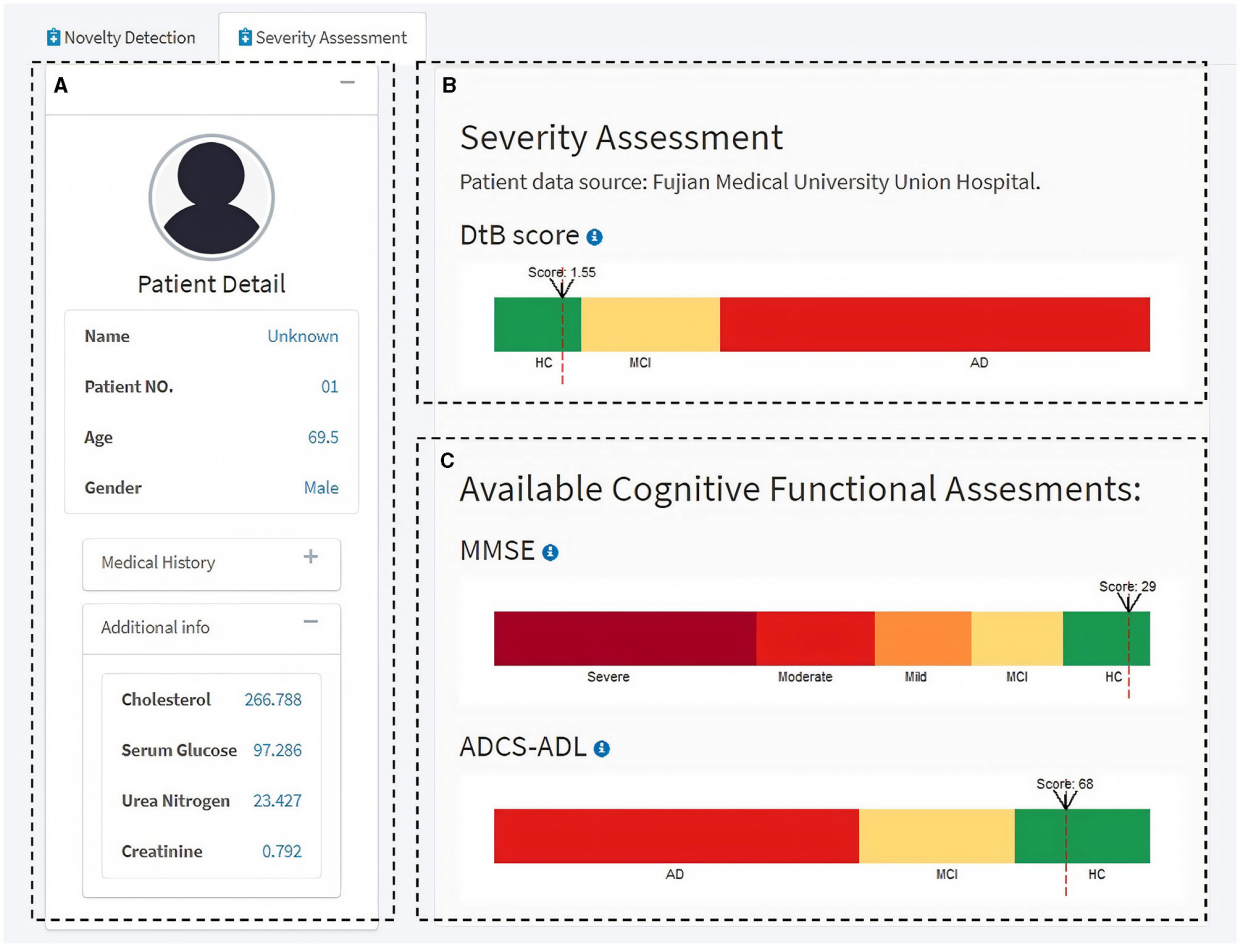
Severity Assessment module of the interactive GUI for CDSS. **(A)** Patient information panel. **(B)** AD severity measurement scale and the AD severity score, i.e., DtB value (red dash line) of the patient. **(C)** Measurement scales for the available CFAs. Additional information can be found through the info icon.

Next, the Severity Assessment module shows an interface of the CDSS for predicting the individual's severity of getting AD (Figure 6). The left side of the module includes the individual's details in a concise format for a quick and unambiguous interpretation (e.g., name, patient no., age, gender, medical history, and some available blood test results) (Figure 6A). The top-right panel visualizes the AD severity measurement (i.e., DtB score) scale and the DtB score of the patient (Figure 6B). Predictions based on the given CFA scores are illustrated in the bottom-right panel (Figure 6C) on a continuous spectrum presented by color progress bars. Intuitively, the AD severity measurement scale is divided into three classes based on the patients' data distribution reflected by DtB values, i.e., HC [CDR = 0; 0 ≤ DtB <*Q*_1−*HC*_ (lower/first quartile of HC)] MCI [CDR = 0.5; *Q*_1−*AD*_(lower/first quartile of AD) ≤ DtB <*Q*_3−*HC*_ (upper/third quartile of HC)], and AD (CDR = 1, 2, or 3; *Q*_3−*HC*_ ≤ DtB ≤ 1.5*IQR* of AD). Note that the color bar of the DtB score has been normalized to the range of 0–10. The CFA score definition and the corresponding cutoff values for disease classification can be found in Tombaugh and McIntyre (1992) and Green et al. (2009). Figure 6C illustrates the manner in which clinicians can be supported in assessing how different CFAs contribute to the predicted DtB score. Overall, this web-based, user-friendly interactive GUI with the built-in ND model demonstrates that our proposed DtB strategy can reflect individuals' severity of developing AD. Corresponding to a large database of existing patient records, the AD severity is evaluated by calculating the DtB score of the data from an undiagnosed patient.

## 4 Discussion

The key uniqueness of this study is the employment of an interpretable ND technique to detect potential MCI/AD. Two distinct cohorts with highly heterogeneous data derived from completely different regions were used for constructing an optimal and closed decision boundary tightly surrounding the HC data, which are solely based on the model training process. The surface area of the decision boundary was minimized to reduce the chance of MCI/AD data acceptance, which enables the boundary to classify whether the unseen data reflects healthy or non-healthy status, depending on the data's relative location to the boundary. Inner data that are close to the boundary indicate a high risk of developing MCI, even if they may be currently detected as HC. For outer data, the closer to the boundary, the milder the cognition declines (e.g., very mild or mild cognitive impairment) they represent; the farther from the boundary, the more severe the cognition declines (e.g., moderate or severe AD) they reflect. This was quantified by our proposed DtB strategy. Our findings also suggest an urgent need for data integration, which should be prioritized by policymakers. Data features involved in this study are multi-modal. The ND models built on different modalities and their combinations were evaluated by three comprehensive metrics.

The ND methods produced comparably high detection performance when only a small subset of the data was used, and this subset was mainly composed of easily accessed CFA. This, once again, highlights that CFA could be a key factor for AD diagnosis in clinical practices (Ding et al., 2018; Bucholc et al., 2019). Our extensive experimental results revealed that models solely using the CFA could produce the best global detection performance (the AUC metric), while those combining IMG would perform better in terms of sensitivity for detecting MCI.

More importantly, our developed GUI integrated two modules (a built-in novelty detector based on existing patient records and AD severity assessment for new patients) and built an overview of a fully functional CDSS. This makes our proposed DtB strategy easier to translate to a clinical domain, which can serve as a supporting tool for clinicians to visually analyze how a different modality or a combination of modalities contributes to predicting AD severity with the basis of given accuracy in detecting MCI/AD against HC.

For a fair comparison, we examined the most developed CDSS typically based on the AIBL data. This examination focused on the research findings published from 1 January 2018 to 31 December 2023 across prominent scholarly databases including the Web of Science, ScienceDirect, IEEE Explore Digital Library, PubMed, and Google Scholar for supplements. To fully cover the relevant studies, the search was performed by utilizing certain keywords followed by AD in the title, abstract, or keywords of the research articles, such as “clinical decision support system,” “diagnosis,” “prognosis,” “artificial intelligence,” and “computerized application.” It is worth noting that only three CDSS studies (Dyrba et al., 2021; Bhattarai et al., 2023; Yi et al., 2023) are associated with AIBL. All of them were developed by using deep learning approaches. Moreover, only one of them (Dyrba et al., 2021) presented an available GUI design. Table 6 details the data description of the three CDSS and ours. Briefly, apart from the study by Bhattarai et al. (2023) which used only non-imaging data, the other two studies and ours included both imaging and non-imaging data. In terms of the development goal (see Table 7), again, apart from the study by Bhattarai et al. (2023) that aimed at medication recommendation for AD patients, the work by Dyrba et al. (2021) includes a binary classification of MCI vs. HC and AD vs. HC, and the work of Yi et al. (2023) focuses on predicting HC-to-MCI and MCI-to-AD. In contrast, our work targets the task of one-class classification for HC vs. MCI vs. AD. Considering the model built in the CDSS, what sets our methodology apart is its strategic reliance on the power of ND, which is a typical unsupervised learning technique and, more importantly, easy to understand and interpret. The three existing CDSS are built using supervised deep-learning techniques that lack model interpretability. The AUC performance comparison shown in the last column of Table 7 evidences that our work is promising for discriminating MCI and AD from HC, which was also confirmed by our clinical consultant.

**Table 6:** Data description of the three AIBL-related CDSS development in comparison to ours.

**Ref**.	**Datasets**	**No. of data samples**	**Data types**
Dyrba et al. (2021)	ADNI^*^-2, 3, GO, AIBL, and DELCODE^*^	ADNI-2/GO: 663 ADNI-3: 575 AIBL: 606 DELCODE: 474	Multi-modal non-imaging data, including demographics, neuroimaging-extracted biomarkers [total intracranial volume (TIV)]; Multi-modal imaging data, including MRI, PET images
Bhattarai et al. (2023)	ADNI and AIBL combined together	1969	Multi-modal non-imaging data, including demographics, CFA (ADAS13, CDRSB, MoCA, MMSE) neuropsychological battery (RAVLT), and neuroimaging-extracted biomarkers (FDG)
Yi et al. (2023)	ADNI, AIBL and GWAS^*^ combined together	1603	Multi-modal non-imaging data, including demographics, CFA (MMSE, ADAS, CDRSB, FAQ, GDS, preclinical Alzheimer's cognitive composite scores), ApoE4 gene type, and neuroimaging-extracted biomarkers (ventricles volume, hippocampus volume, WBV, entorhinal volume, fusiform volume, middle temporal gyrus volume, TIV); Single modal imaging data, i.e., MRI images
Ours	AIBL and FMUUH	AIBL: 1487 FMUUH: 330	Multi-modal imaging and non-imaging data, see Table 1 for more details

^*^ADNI is the Alzheimer's Disease Neuroimaging Initiative; DELCODE is the German Center for Neurodegenerative Diseases multicenter observational study on Longitudinal Cognitive Impairment and Dementia; and GWAS is the genome-wide association study.

**Table 7:** Performance comparison of the data modeling methods in the compared CDSS development and ours.

**Ref**.	**Data modeling approach**	**Development goal**	**AUC performance**
Dyrba et al. (2021)	Convolutional Neural Networks (supervised learning)	Binary classification of MCI vs. HC and AD vs. HC	0.763, 0.684, and 0.775 for MCI vs. HC in AIBL, ADNI-3, and DELCODE 0.95, 0.913, and 0.953 for AD vs. HC in AIBL, ADNI-3, and DELCODE
Bhattarai et al. (2023)	Reinforcement learning (supervised learning)	Medication recommendation for AD patients	Not applicable
Yi et al. (2023)	Deep learning-based survival clustering (supervised learning)	Prediction of HC-to-MCI and MCI-to-AD	0.708, 0.802, 0.876, and 0.886 for HC-to-MCI in 1, 3, 5, and 10 years; 0.81, 0.914, 0.957, and 0.979 for MCI-to-AD in 1, 3, 5, and 10 years
Ours	MoG-based ND algorithms (unsupervised learning)	One-class classification of HC vs. MCI vs. AD	0.8757 for HC vs. MCI vs. AD in AIBL; 0.9443 for HC vs. MCI vs. AD in FMUUH

Considering the two datasets employed in this study, even though they are from different regions of Australia and China, the similarities between them are: (1) the proportion of female is higher than that of male in both HC and AD; (2) the relative proportion of female/male is similar for both categories; and (3) the mean age of AD is higher than that of HC. The only difference lies in the younger subjects in FMUUH compared to those in AIBL. For a fair comparison, Table 8 lists the proportion of females/males and the mean age along with the standard deviation (STD) for HC and AD categories that were available in both datasets. It is worth noting that our findings from the proposed ND framework on both data are consistent, indicating that the MoG-based ND method applied to the same single modality (i.e., CFA, even including feature variations) achieved the highest overall performance in detecting MCI and AD. As such, our interactive GUI, tailored for non-technical stakeholders incorporating the proposed ND framework, is generalizable and adaptive according to the two employed cohorts.

**Table 8:** The comparison of demographic features between AIBL and FMUUH.

**Data**	**Gender (F/M)**		**Age (Mean** ±**STD)**	
	**HC**	**AD**	**HC**	**AD**
AIBL	54.6%/45.4%	63%/37%	73.3 ± 6.9	75.6 ± 7.9
FMUUH	59.5%/40.5%	61%/39%	63.3 ± 6.6	69.4 ± 8.5

F, Female; M, Male; STD, standard deviation.

On the other hand, this study has several limitations worth noting that could guide future extensions and improvements. First, we simply ignored missing data. An approach for missing data imputation is currently being developed, which will be incorporated into the system later. Second, we only selected the top ten features from the AIBL data that were significant to CDR and used three different univariate filters to rank the feature importance. Such filtering approaches may lead to the loss of relevant features that are meaningless by themselves but crucial to model improvement when considered together. To tackle this deficiency, we previously applied wrapper methods to evaluate the importance of specific feature sets (Bucholc et al., 2019). Work is currently being done to improve our ND model by developing a wrapper that can obtain a subset of better-integrated data from different modalities. Third, to get a large data size, we integrated the multi-modal AIBL data collected at different time points together. Repeat visitors who participated in the AIBL study were considered as different visitors. However, some modalities, such as medical history, ApoE genotype, and gender, were not time-evolved. This may be the reason for the poor performance when the models were trained by the two modalities of MH and DEM and BLO and ApoE. We are currently in the process of conducting further investigations for the ND technique on larger-sized data [e.g., ADNI (The Alzheimer's Disease Neuroimaging Initiative) (Mueller et al., 2005) or NACC (The National Alzheimer's Coordinating Center) (Beekly et al., 2007) data] and integrating more FMUUH data from the local hospital. Additionally, the current study and our previous development of a CDSS prototype using other machine learning approaches (Bucholc et al., 2019) have provided a solid foundation for the next extension phase to develop a CDSS employing the ND technique. Furthermore, in our previous work (Ding et al., 2015), we had proposed a new ND approach, namely level set boundary description (LSBD). Being superior to the traditional ND methods (e.g., based on probability, distance, clustering, statistics, and support vector machines), the LSBD introduced some interesting properties for boundary construction, such as non-linear problem addressable without using a kernel trick, non-parametric, dynamically time-evolved to better fit the data distribution, boundary shape easily manageable, and straightforward implemented in the given data space. Therefore, based on the current study, we will deeply investigate LSBD for early MCI and AD discrimination from HC populations. Finally, more development could be carried out in the current GUI for a fully functional CDSS, including local data collection (data input module), options for choosing different ND algorithms, more scales of AD severity according to the five CDR categories with more available data, and so on.

## 5 Conclusion

This study first utilized four representative and easily interpretable ND algorithms to build novelty detectors based on heterogeneous Alzheimer's datasets from different regions. The intrinsic pattern behind AD was investigated in the distinct cohort study by comparing the performance of models trained on different modalities and different combinations of modality types. We found that the best overall performance could be obtained when only CFA features were used. Hence, applying some non-invasive and easily accessible features can significantly detect cognitive decline at an early stage. Although this finding has been reported in our previous contributions (Ding et al., 2018; Bucholc et al., 2019), the uniqueness of this study is that we first utilized ND in the area and then proposed a DtB strategy to quantitatively discriminate MCI/AD from HC. More importantly, the training of the ND model with the built-in DtB strategy is solely based on HC data, which are more easily and less costly to obtain than MCI/AD data, unlike traditional methods that require labeled and balanced data from both HC and non-HC for model training.

Crucially, the insight of the study was presented by the proposed DtB strategy by illustrating and quantifying the decision boundary along with data distribution. The strategy could intuitively and objectively reflect individuals' severity of developing AD. More practically, the GUI we developed offers a translational and interactively visual tool even for those lacking an IT background and experience in AD recognition. These results would help inform future guidelines for the development of an integrated functional CDSS aimed at early-stage diagnosis for MCI/AD.

## Data availability statement

The data analyzed in this study is subject to the following licenses/restrictions: Data sharing agreement. Requests to access these datasets should be directed to AIBL dataset: https://aibl.csiro.au/; FMUUH data: email unionqyye@163.com (QY) or xiaoxinskyhost@163.com (JX).

## Ethics statement

The studies involving humans were approved by Fujian Medical University Union Hospital Ethics Committee. The studies were conducted in accordance with the local legislation and institutional requirements. Written informed consent for participation in this study was provided by the participants' legal guardians/next of kin.

## Author contributions

HY: Project administration, Supervision, Writing – review & editing. JM: Methodology, Software, Visualization, Writing – original draft. QY: Data curation, Resources, Validation, Writing – review & editing. MB: Writing – review & editing. SL: Methodology, Software, Writing – review & editing. WG: Writing – review & editing. JP: Investigation, Writing – review & editing. JX: Data curation, Resources, Validation, Writing – review & editing. XD: Conceptualization, Formal analysis, Methodology, Project administration, Supervision, Visualization, Writing – review & editing.
