# Supplementary material for: Distance-based novelty detection model for identifying individuals at risk of developing Alzheimer's disease
Source: Front Aging Neurosci. 2024 Apr 15;16:1285905. doi: 10.3389/fnagi.2024.1285905 (PMC11057441; doi:10.3389/fnagi.2024.1285905)
Supplement: Supplementary file 1 [file Data_Sheet_1.DOCX]

***Supplementary Material***

# Supplementary Figures and Tables

## Supplementary Figures


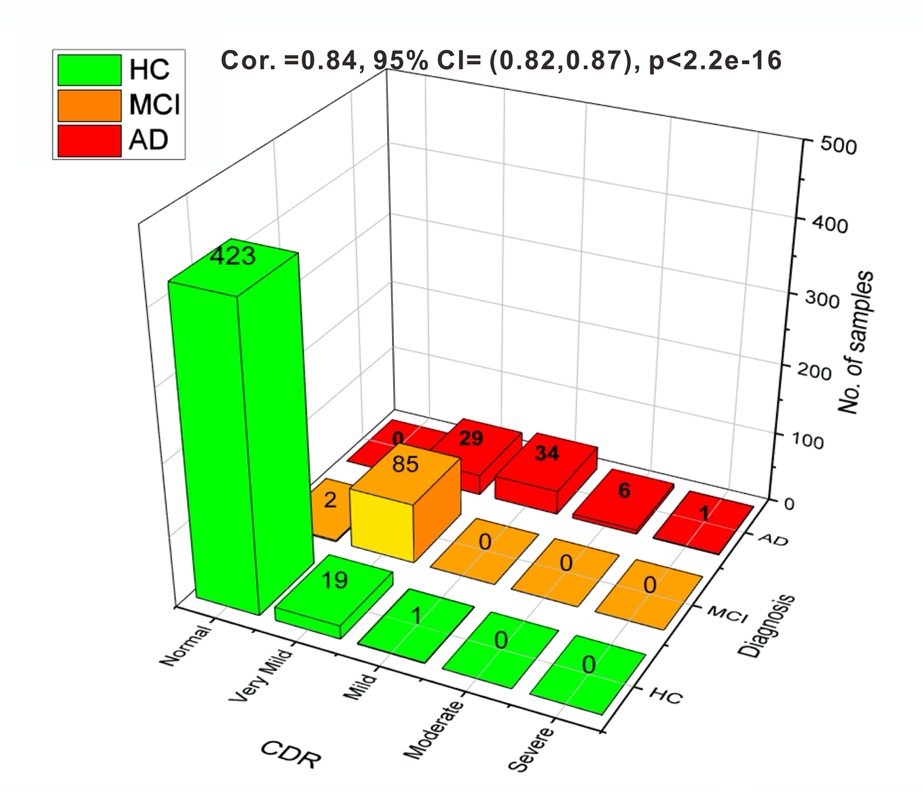


**Supplementary Figure 1.** Strong correlation between clinical diagnosis and Clinical Dementia Rating (CDR) categories. Green bars: healthy control (HC). Orange bars: MCI. Red bars: AD. Cor.: Pearson correlation coefficient; CI: confidence interval. CDR scores reflect 5 categories: healthy controls (CDR=0), very mild (CDR=0.5), mild (CDR=1), moderate (CDR=2), and severe (CDR=3) patients. Clinical diagnosis contains 3 categories: HC, MCI, and AD. The data distribution along with the Cor., 95% CI, and p-value showed a significant correlation between clinical diagnosis and CDR.


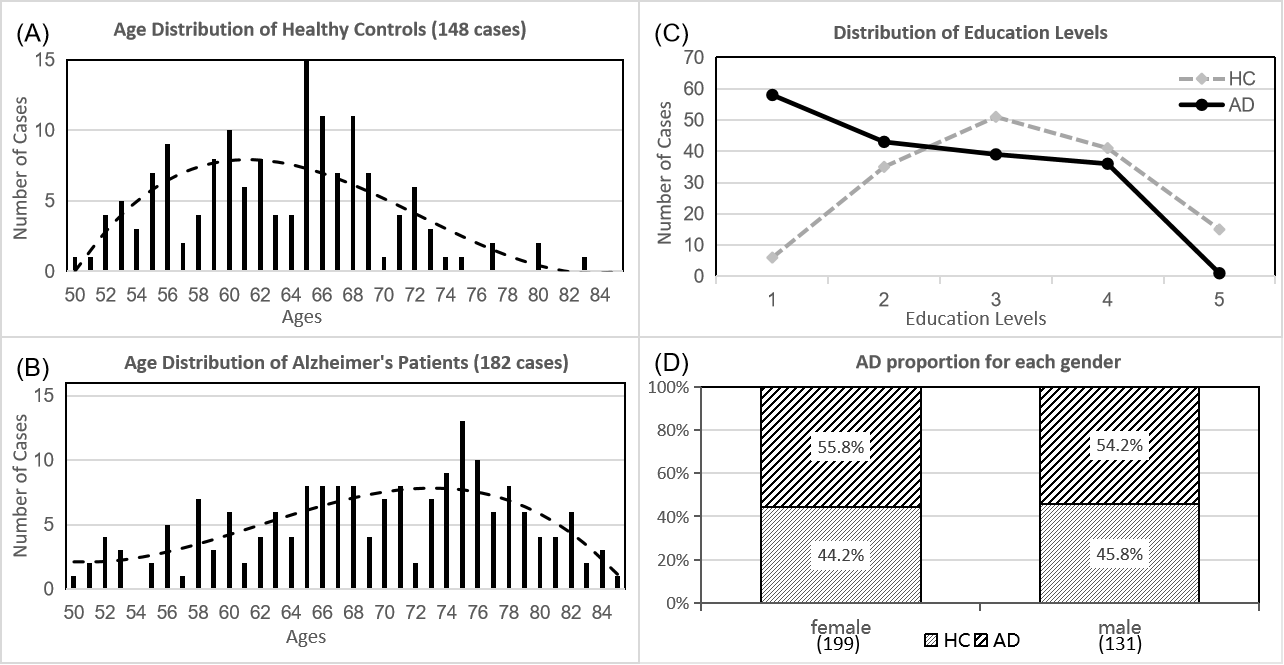


**Supplementary Figure 2.** Demographic features distribution of the FMUUH data. (A). Age distribution in HC groups where people at risk of AD are mainly between 65 to 69. (B). Age distribution in AD groups is mainly between 74 and 78. (C). Distribution of Education levels. The number of AD cases increases as the education level declines, especially when the education level was illiteracy (Education level = 1), the number of AD was about 8.7 times more than that of HC (Education Level - 1: Illiteracy; 2: Primary school; 3: Junior high school; 4: High school/Technical secondary school; 5= University/College). (D). AD proportion for each gender. Despite of more females than males with a total number of 199 and 131, the proportion of AD in each gender is similar, 55.8% and 54.2%, respectively.


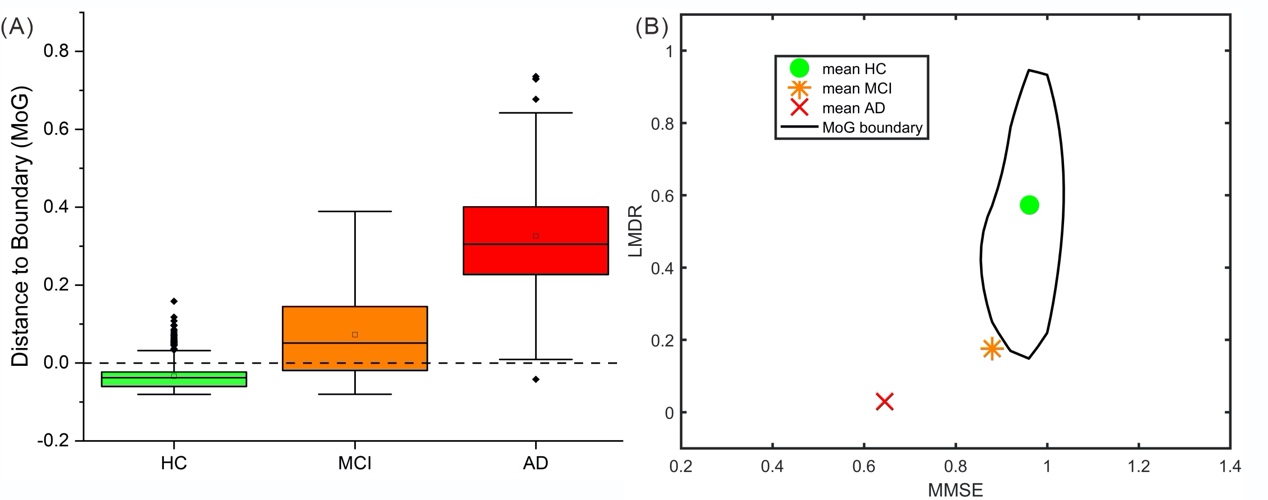


**Supplementary Figure 3.** Differentiation of HC, MCI and AD based on the proposed DtB strategy. (A) Box plot of the DtB values between the AIBL data and the ND boundary generated by MoG. (B) The decision boundary generated by the MoG method. The positions of mean HC/MCI/AD are represented by the green dot, orange star, and red cross, respectively. The closed black curve is the decision boundary.

## Supplementary Tables

**Supplementary Table 1.** Demographic distribution of AIBL data.

| Clinical diagnosis | CDR | Number | Gender(M/F) | Age (Mean±STD) |
| --- | --- | --- | --- | --- |
| HC | 0 | 989 | 449/540 | 73.3±6.9 |
| MCI | 0.5 | 493 | 195/144 | 75.5±7.5 |
| AD | 1, 2, and 3 | 154 | 57/97 | 75.6±7.9 |

**Supplementary Table 2.** The performance of the ND models using all possible AIBL modality combinations (①: CFA; ②: IMG; ③: MH&DEM; ④: BLO&ApoE.) after feature selection.

| Modality combinations |  | False rejection rate : 0~1  AUC (95%CI) | False rejection rate = 0.1 | | |
| --- | --- | --- | --- | --- | --- |
|  |  |  | Specificity | Sensitivity | |
|  |  |  | HC | MCI | AD |
| ①②③④ | KNN | **0.8690 (0.8240-0.9140)** | 89.73% | 62.64% | 92.82% |
|  | MoG | 0.8552 (0.8185-0.8919) | 85.89% | 64.55% | 92.82% |
|  | KMEANS | 0.8676 (0.8179-0.9173) | 87.43% | 58.02% | 97.39% |
|  | SVDD | 0.8462 (0.7636-0.9288) | 88.28% | 51.73% | 89.54% |
| ①②③ | KNN | **0.8405 (0.7443-0.9366)** | 90.20% | 51.35% | 92.68% |
|  | MoG | 0.8374 (0.7388-0.9360) | 85.60% | 60.03% | 93.08% |
|  | KMEANS | 0.7867 (0.6587-0.9147) | 87.38% | 18.47% | 99.60% |
|  | SVDD | 0.8205 (0.6703-0.9707) | 87.57% | 52.78% | 99.60% |
| ①②④ | KNN | 0.8232 (0.7440-0.9023) | 90.37% | 64.10% | 94.62% |
|  | MoG | **0.8416 (0.7224-0.9607)** | 89.68% | 57.80% | 90.12% |
|  | KMEANS | 0.8394 (0.7533-0.9254) | 90.16% | 60.62% | 99.43% |
|  | SVDD | 0.8315 (0.7161-0.9468) | 85.27% | 60.05% | 97.18% |
| ①③④ | KNN | 0.8000 (0.7127-0.8873) | 89.59% | 54.84% | 91.89% |
|  | MoG | 0.8266 (0.7624-0.8908) | 86.04% | 59.14% | 91.89% |
|  | KMEANS | 0.8257 (0.7329-0.9184) | 90.59% | 52.08% | 86.69% |
|  | SVDD | **0.8304 (0.6963-0.9644)** | 86.50% | 56.28% | 93.80% |
| ②③④ | KNN | 0.6948 (0.5944-0.7953) | 90.67% | 13.97% | 33.33% |
|  | MoG | **0.7109 (0.6132-0.8086)** | 89.42% | 25.04% | 47.17% |
|  | KMEANS | 0.6971 (0.6480-0.7462) | 83.79% | 25.65% | 45.50% |
|  | SVDD | 0.6769 (0.6092-0.7446) | 87.80% | 20.51% | 37.00% |
| ①③ | KNN | 0.7858 (0.6710-0.9005) | 89.04% | 53.68% | 86.84% |
|  | MoG | **0.8048 (0.6880-0.9214)** | 89.02% | 47.53% | 75.43% |
|  | KMEANS | 0.7452 (0.6686-0.8217) | 84.08% | 52.55% | 66.72% |
|  | SVDD | 0.7783 (0.6646-0.8919) | 86.21% | 63.93% | 83.99% |
| ①④ | KNN | 0.7926 (0.7439-0.8414) | 91.31% | 33.48% | 87.47% |
|  | MoG | **0.8113 (0.7085-0.9141)** | 91.33% | 38.39% | 91.44% |
|  | KMEANS | 0.8058 (0.7435-0.8682) | 91.58% | 41.32% | 87.47% |
|  | SVDD | 0.8063 (0.7396-0.8729) | 90.62% | 39.90% | 93.17% |
| ②③ | KNN | **0.7745 (0.6859-0.8632)** | 90.61% | 37.95% | 66.44% |
|  | MoG | 0.7057 (0.5513-0.8601) | 78.68% | 45.77% | 65.97% |
|  | KMEANS | 0.6988 (0.5429-0.8547) | 71.21% | 59.84% | 64.73% |
|  | SVDD | 0.6355 (0.5772-0.6938) | 87.28% | 23.41% | 27.16% |
| ②④ | KNN | 0.6696 (0.4809-0.8583) | 89.02% | 23.60% | 44.68% |
|  | MoG | **0.6783 (0.5316-0.8251)** | 89.12% | 24.71% | 46.44% |
|  | KMEANS | 0.6691 (0.6179-0.7203) | 83.32% | 29.83% | 50.94% |
|  | SVDD | 0.6553 (0.5650-0.7457) | 88.08% | 19.54% | 27.76% |
| ③④ | KNN | **0.6103 (0.5517-0.6689)** | 89.45% | 16.78% | 25.43% |
|  | MoG | 0.5904 (0.4639-0.7169) | 68.06% | 41.07% | 50.20% |
|  | KMEANS | 0.5824 (0.4601-0.7047) | 64.58% | 42.09% | 31.01% |
|  | SVDD | 0.5275 (0.4042-0.6507) | 86.29% | 16.10% | 14.92% |

**Supplementary Table 3.** The performance of ND models using all possible modality combinations without feature selection (①: CFA; ②:IMG; ③:MH&DEM; ④: BLO&ApoE).

| Modality combinations |  | False rejection rate : 0~1 | False rejection rate = 0.1 | | |
| --- | --- | --- | --- | --- | --- |
|  |  | AUC (95%CI) | Specificity | Sensitivity | |
|  |  |  | HC | MCI | AD |
| ①②③④ | KNN | **0.8479 (0.7897-0.9061)** | 90.79% | 30.25% | 38.59% |
|  | MoG | 0.7890 (0.6610-0.9169) | 82.12% | 43.24% | 88.47% |
|  | KMEANS | 0.7551 (0.6924-0.8178) | 67.52% | 61.78% | 83.57% |
|  | SVDD | 0.6856 (0.5604-0.8107) | 77.65% | 59.26% | 73.68% |
| ①②③ | KNN | **0.8579 (0.7510-0.9649)** | 89.62% | 50.07% | 92.59% |
|  | MoG | 0.8003 (0.6951-0.9056) | 84.24% | 38.02% | 79.98% |
|  | KMEANS | 0.7952 (0.6918-0.8985) | 71.48% | 73.81% | 89.38% |
|  | SVDD | 0.7098 (0.6144-0.8052) | 81.11% | 55.98% | 85.22% |
| ①②④ | KNN | **0.8239 (0.7422-0.9055)** | 90.03% | 47.44% | 82.97% |
|  | MoG | 0.8153 (0.6831-0.9474) | 86.42% | 50.36% | 92.81% |
|  | KMEANS | 0.8149 (0.7283-0.9015) | 89.99% | 47.36% | 81.39% |
|  | SVDD | 0.8161 (0.7142-0.9180) | 89.42% | 50.65% | 82.68% |
| ①③④ | KNN | **0.8427 (0.8018-0.8836)** | 89.43% | 38.67% | 66.64% |
|  | MoG | 0.7735 (0.6758-0.8713) | 82.68% | 36.02% | 88.97% |
|  | KMEANS | 0.7557 (0.6757-0.8357) | 67.31% | 66.66% | 89.43% |
|  | SVDD | 0.6805 (0.6066-0.7543) | 76.96% | 52.01% | 84.69% |
| ②③④ | KNN | **0.8113 (0.7402-0.8825)** | 90.87% | 27.98% | 45.77% |
|  | MoG | 0.6981 (0.5888-0.8074) | 72.94% | 42.32% | 55.64% |
|  | KMEANS | 0.7113 (0.6213-0.8014) | 74.01% | 53.98% | 76.40% |
|  | SVDD | 0.5476 (0.3771-0.7181) | 77.40% | 34.61% | 38.00% |
| ①② | KNN | 0.8464 (0.7380-0.9548) | 91.40% | 68.61% | 92.27% |
|  | MoG | 0.8558 (0.7717-0.9400) | 90.69% | 69.86% | 95.85% |
|  | KMEANS | **0.8564 (0.7492-0.9636)** | 90.71% | 67.60% | 96.87% |
|  | SVDD | 0.8442 (0.7190-0.9694) | 88.99% | 68.57% | 92.72% |
| ①③ | KNN | **0.8248 (0.7707-0.8789)** | 88.65% | 46.57% | 90.79% |
|  | MoG | 0.7666 (0.6587-0.8744) | 88.81% | 19.43% | 89.29% |
|  | KMEANS | 0.7396 (0.6684-0.8107) | 79.31% | 60.19% | 90.90% |
|  | SVDD | 0.6951 (0.6249-0.7652) | 81.40% | 53.29% | 89.82% |
| ①④ | KNN | 0.7877 (0.7286-0.8469) | 89.18% | 43.17% | 84.64% |
|  | MoG | 0.7998 (0.7236-0.8760) | 85.71% | 45.09% | 93.31% |
|  | KMEANS | **0.8052 (0.7315-0.87893)** | 88.04% | 42.31% | 84.02% |
|  | SVDD | 0.7949 (0.7188-0.8709) | 89.60% | 48.91% | 84.69% |
| ②③ | KNN | **0.8191 (0.7522-0.8860)** | 87.77% | 38.24% | 68.89% |
|  | MoG | 0.6807 (0.5787-0.7826) | 67.67% | 62.47% | 94.43% |
|  | KMEANS | 0.7339 (0.6434-0.8243) | 76.85% | 66.54% | 78.23% |
|  | SVDD | 0.5674 (0.4170-0.7177) | 77.96% | 28.59% | 42.94% |
| ②④ | KNN | **0.7077 (0.6124-0.8030)** | 91.76% | 28.13% | 45.80% |
|  | MoG | 0.6941 (0.6254-0.7629) | 85.92% | 28.70% | 44.73% |
|  | KMEANS | 0.6437 (0.5909-0.6965) | 74.79% | 39.98% | 58.23% |
|  | SVDD | 0.6531 (0.5692-0.7370) | 90.68% | 24.07% | 24.80% |
| ③④ | KNN | **0.7954 (0.7647-0.8261)** | 88.93% | 36.15% | 48.17% |
|  | MoG | 0.6364 (0.5449-0.7279) | 63.13% | 66.05% | 69.66% |
|  | KMEANS | 0.7045 (0.6505-0.7586) | 68.19% | 64.04% | 71.22% |
|  | SVDD | 0.5110 (0.4279-0.5942) | 77.57% | 30.87% | 30.15% |
| ① | KNN | 0.8521 (0.7250-0.9792) | 89.40% | 59.87% | 96.79% |
|  | MoG | **0.8757 (0.7982-0.9532)** | 89.63% | 67.33% | 96.79% |
|  | KMEANS | 0.8527 (0.7405-0.9650) | 89.11% | 56.92% | 95.23% |
|  | SVDD | 0.8267 (0.7013-0.9521) | 84.94% | 60.83% | 98.43% |
| ② | KNN | **0.7147 (0.6549-0.7745)** | 90.38% | 33.45% | 54.58% |
|  | MoG | 0.6984 (0.6551-0.7419) | 84.81% | 37.85% | 65.39% |
|  | KMEANS | 0.7107 (0.6559-0.7654) | 89.72% | 32.01% | 74.58% |
|  | SVDD | 0.6903 (0.6285-0.7520) | 90.69% | 32.06% | 55.74% |
| ③ | KNN | **0.6868 (0.4992-0.8743)** | 89.31% | 34.52% | 61.78% |
|  | MoG | 0.5938 (0.4076-0.7800) | 73.40% | 41.87% | 53.42% |
|  | KMEANS | 0.6403 (0.4791-0.8015) | 74.24% | 36.07% | 56.42% |
|  | SVDD | 0.5070 (0.2995-0.7146) | 76.45% | 33.27% | 42.53% |
| ④ | KNN | **0.6088 (0.5081-0.7094)** | 89.55% | 24.34% | 30.75% |
|  | MoG | 0.5920 (0.5394-0.6446) | 89.10% | 21.27% | 23.23% |
|  | KMEANS | 0.5742 (0.4933-0.6550) | 69.65% | 37.72% | 54.63% |
|  | SVDD | 0.5706 (0.5154-0.6257) | 90.53% | 10.30% | 12.63% |

# The computational details of the ND methods

## KNN

The KNN is a representative distance-based ND method assuming that all normal data points are close to each other and anomalies are far from the normal points [1].The KNN method first calculates the distance between the data point x and its k nearest neighbors (denoted as ${NN}_{k}$(x)) and then calculates the distance from these nearest neighbor ${NN}_{k}$(x) to their k nearest neighbors ${NN}_{k}$(${NN}_{k}$(x)).Finally, it discriminates whether a data point x is normal or abnormal by comparing these two distances. The acceptance function for a test data point can be defined as [1]:

$$f_{KNN}\left( x \right)=I(\frac{\left\| x-{NN}_{k}(x) \right\|}{\left\| {NN}_{k}\left( x \right)-{NN}_{k}{NN}_{k}\left( x \right) \right\|}\leq1)$$

Where $I(\cdot)$ is a logical indicator function. If $\cdot$ is true, then $I\left( \cdot\right)=1$ indicates x normal; otherwise $I\left( \cdot\right)=0$ indicates x abnormal. $\left\| \cdot\right\|$ represents the Euclidean distance. The *k* is the parameter to be optimized in KNN.

## MoG

The MoG is a commonly used density-based ND method by calculating a linear combination of N components of normal distribution on the given data [2].The probability density of data x can be estimated with [2]:

$$P_{MoG}\left( x \right)=\frac{1}{N}\sum_{j=1}^{N} \left\{ a_{j}\frac{1}{{(2\pi)}^{\frac{d}{2}}\left| \sum j \right|^{\frac{1}{2}}}exp\left\{ -\frac{1}{2}{(x-\mu_{j})}^{T}\sum_{j}^{-1} (x-\mu_{j}) \right\} \right\}$$

where $a_{j}$ is the mixture coefficients, $\mu_{j}$is the mean of the j^th^ Gaussian component, and $\sum j$is the covariance matrix. Data lying in a high-density area are accepted as normal; otherwise, are detected as abnormal. The *N* is the parameter to be optimized in MoG.

## KMEANS

KMEANS, a representative clustering-based ND method, is one of the most popular techniques due to its simplicity of implementation [3].This method clusters normal data using a small number (i.e., k) of prototypes. The centroids of k clustered prototypes are optimized by the following minimized square error:

$${error}_{KMEANS}=\sum_{i} {min}_{k}\left\| x_{i}-\mu_{k} \right\|^{2}$$

Where $\mu_{k}$ is the centroid associated with the *k*^th^ cluster. Any data excluded by all clusters would be detected abnormal. The *k* is the parameter to be optimized in KMEANS.

## SVDD

The SVDD represents a support vector machine-based ND method (Tax and Duin, 2004). It employs a hypersphere to define a closed decision boundary around normal data. The general formulation is based on the following relations [4]:

$$S=\left\{ \left( x_{i},x_{j} \right) | x_{i},x_{j}\in same class \right\}$$

$$D=\left\{ \left( x_{i},x_{j} \right) | x_{i},x_{j}\in different classes \right\}$$

where *S* is a set of similar examples from the same class, while the *D* includes those that are dissimilar from different classes. The learning process involves minimizing the distances between each pair of data point in *S* and maximizing in *D*. The radius of the hypersphere **R** can be calculated by the distance between center and one of the unbounded support vectors $x_{S}$:

$$R^{2}=1-2\sum_{i} a_{i}K\left( x_{i},x_{s} \right)+\sum_{i,j} a_{i}a_{j}K\left( x_{i},x_{j} \right)$$

where $x_{i},x_{j}$ are the *i^th^* and *j^th^* data point in training set. And $a$ is the Lagrange multiplier with $\sum a=1 and 0\leq a\leq C$, where $C$ is the penalty weight that controls the trade-off between the fraction of rejected normal data and the volume of the hypersphere. In this study, the radial basis kernel [4], which is the selected kernel, is given by:

$$K\left( x_{i},x_{j} \right)=exp(\frac{-\left\| x_{i}-x_{j} \right\|^{2}}{\sigma^{2}})$$

where σ represents the kernel parameter (width) to be optimized in SVDD.

[1] V. Hautamaki, I. Karkkainen, and P. Franti, "Outlier detection using k-nearest neighbour graph," in Proceedings of the 17th International Conference on Pattern Recognition, 2004. ICPR , vol. 3, pp. 430-433 Vol.3, doi: 10.1109/ICPR.2004.1334558.

[2] C. Bishop, Pattern Recognition and Machine Learning. Springer, 2006.

[3] S. Chawla and A. Gionis, "k-means–: A unified approach to clustering and outlier detection," in Proceedings of the 2013 SIAM International Conference on Data Mining (SDM), pp. 189-197.

[4] A. Lazzaretti and D. Tax, An Adaptive Radial Basis Function Kernel for Support Vector Data Description. 2015, pp. 103-116, doi: 10.1007/978-3-319-24261-3_9
